# Constructing Desired Vertical Component Distribution Within a PBDB-T:ITIC-M Photoactive Layer via Fine-Tuning the Surface Free Energy of a Titanium Chelate Cathode Buffer Layer

**DOI:** 10.3389/fchem.2018.00292

**Published:** 2018-08-20

**Authors:** Yiming Bai, Bo Yang, Xiaohan Chen, Fuzhi Wang, Tasawar Hayat, Ahmed Alsaedi, Zhan'ao Tan

**Affiliations:** ^1^State Key Laboratory of Alternate Electrical Power System with Renewable Energy Sources, North China Electric Power University, Beijing, China; ^2^Beijing Key Laboratory of Energy Safety and Clean Utilization, North China Electric Power University, Beijing, China; ^3^Department of Mathematics, Quiad-I-Azam University, Islamabad, Pakistan; ^4^NAAM Research Group, Faculty of Science, King Abdulaziz University, Jeddah, Saudi Arabia

**Keywords:** polymer solar cells, vertical component distribution, surface free energy, cathode buffer layer, annealing temperature

## Abstract

Rationally controlling the vertical component distribution within a photoactive layer is crucial for efficient polymer solar cells (PSCs). Herein, fine-tuning the surface free energy (SFE) of the titanium(IV) oxide bis(2,4-pentanedionate) (TOPD) cathode buffer layer is proposed to achieve a desired perpendicular component distribution for the PBDB-T:ITIC-M photoactive layer. The Owens-Wendt method is adopted to precisely calculate the SFE of TOPD film jointly based on the water contact angle and the diiodomethane contact angle. We find that the SFE of TOPD film increases as the annealing temperature rises, and the subtle SFE change causes the profound vertical component distribution within the bulk region of PBDB-T:ITIC-M. The results of secondary-ion mass spectroscopy visibly demonstrate that the TOPD film with an SFE of 48.71 mJ/cm^2^, which is very close to that of the ITIC film (43.98 mJ/cm^2^), tends to form desired vertical component distribution. Consequently, compared with conventional bulk heterojunction devices, the power conversion efficiency increases from 9.00 to 10.20% benefiting from the short circuit current density increase from 14.76 to 16.88 mA/cm^2^. Our findings confirm that the SFE adjustment is an effective way of constructing the desired vertical component distribution and therefore achieving high-efficiency PSCs.

## Introduction

Stimulated by the need for a clean renewable energy source, there has been considerable interest in exploring polymer solar cells (PSCs) due to their unique properties of low cost, light weight, and flexibility (Krebs et al., 2010; Li G. et al., 2012; Zhao et al., 2017). The state-of-the-art device configuration is the sandwich bulk heterojunction (BHJ), blending the conjugated polymer donor compactly with a fullerene or fullerene-free acceptor (Ouyang et al., 2015; Jiang et al., 2017; Peng et al., 2018). Very recently, the best power conversion efficiency (PCE) of single-junction PSCs based on the fullerene acceptor has exceeded 11.7% (Zhao et al., 2016), and that of fullerene-free PSCs has reached up to 14%. This can be attributed to the development of new electron donors and the matching acceptors, device structures and novel interfacial layers (Xiao et al., 2017).

The BHJ photoactive layer, as the main functional layer for light absorption, exciton generation, dissociation, and transportation, is commonly fabricated by spin-coating the mixed solution of electron donors and acceptors (Heriot and Jones, 2005; Lu et al., 2013; Xie et al., 2014; Bin et al., 2016). The mixed components will form a vertical phase separated photoactive layer during the film-drying process, which ensures a large interfacial area for efficient exciton dissociation and facilitates the charge transportation and selective collection via the formation of bi-continuous interpenetrating networks (Qiu et al., 2008; Xu et al., 2010; Meier et al., 2011). Hence, an in-depth understanding about the vertical component distribution within the photoactive layer is absolutely imperative for realizing efficient PSCs. However, its mechanism is complex and still vague, which involves thermodynamics, dynamics, free-surface, and interface effects during the blend formation process (Kim et al., 2017).

A number of studies on fullerene PSCs have illustrated that the perpendicular component distribution of photoactive blends is greatly influenced by the processing conditions, such as solvent soaking, solvent flush treatment, and solvent additives (Li C. Z. et al., 2012; Heo et al., 2014; Van Franeker et al., 2015). Li et al. introduced a mixed solvent-soaking approach to obtain an interpenetrating network composed of highly crystalline regioregular poly(3-hexylthiophene) (P3HT) and [6,6]-phenyl-C_61_-butyric acid methyl ester (PC_61_BM) nano-aggregates (Hui et al., 2011). The 2-chlorophenol flush treatment is also a simple and feasible way to acquire an optimal vertical composition profile of photoactive blends, leading to an increased PCE from 6.18 to 10.15% for inverted PSCs based on poly[4,8-bis(5-(2-ethylhexyl)thiophen-2-yl)benzo[1,2-b:4,5-b0]dithiophenealt-3-fluorothieno[3,4-b]thiophene-2-carboxylate] (PTB7-Th):[6,6]-phenyl-C_71_-butyric acid methyl ester (PC_71_BM) blends (Wang et al., 2016). The solvent additive 1,8-diiodooctane (DIO) with high boiling point, using the solubility of the different components to change and affect the donor and acceptor phase distributions, can selectively dissolve PCBM and facilitate long-range diffusion of PCBM to form a bicontinuous pathway for electron and hole transportation at the latter stage of the film-drying process (Xiao et al., 2014). However, few investigations have been conducted to elucidate the influence of processing parameters on the perpendicular component distribution of fullerene-free photoactive blends though it well exists, and this is an area warranting urgent exploration (Yan et al., 2017).

Evidence also shows that the free energy of electron donors and acceptors, and the substrate surface onto which they are deposited has a direct impact on the perpendicular component distribution (Jones et al., 1989; Jasieniak et al., 2016). Kim et al. found that P3HTs with hydroxyl-, ethyl-, perfluoro-, and bromo- end groups have different surface free energies (SFEs), and the vertical stratification in their blends with PCBM can be tuned as the surface energy difference between electron donors and acceptors (Kim et al., 2010). It is well-known that the system is the most stable if and only if the systematic energy is the minimum. Jones put forward that any surface energy difference between the pure components allows the photoactive blends to minimize its total free energy by increasing the surface concentration of the low-energy component (Jones et al., 1989). In contrast, Bjöström and Tillack believed that the variation of the substrate SFE affects the perpendicular component distribution within the bulk region rather than the air surface region (Björström et al., 2005; Tillack et al., 2011). Germack found that for the similar P3HT:PCBM blends (the surface energies for polymers P3HT and PCBM are about 23 and 45 mJ/m^2^, respectively), their vertical component distributions are influenced by the substrates (Germack et al., 2009). Namely, if the P3HT:PCBM blends are deposited on poly(3,4-ethylenedioxythiophene):poly(styrenesulfonate) (PEDOT:PSS) with a surface energy of 45 mJ/m^2^, the PCBM is enriched near the substrate surface, while the P3HT is enriched near the substrate interface as well as the free surface if the P3HT:PCBM blends are deposited on a poly(thienothiophene):Nafion substrate with a surface energy of 23 mJ/m^2^ (Germack et al., 2010). The aforementioned reports are helpful for constructing ideal vertical morphology and further achieving efficient PSCs. However, most of the studies primarily focus on fullerene PSCs, and few works are available on fullerene-free PSCs, especially on finding modulation approaches to realize the desired vertical phase separation.

Hence, the present work is aimed at exploring the influence of processing parameters on the SFE of titanium(IV) oxide bis(2,4-pentanedionate) (TOPD) cathode buffer layer and further elucidating the impact of SFE on the vertical component dis-tribution within the (poly[(2,6-(4,8-bis(5-(2-ethylhexyl)thiophen-2-yl)benzo[1,2-b:4,5-b′]dithiophene)-co-(1,3-bis(5-thiophene-2-yl)-5,7-bis(2-ethylhexyl)benzo[1,2-c:4,5-c]dithiophene-4,8-dione)] PBDB-T:ITIC-M (3,9-bis((Z)-1-(6-(dicyanomethylene)-2-methyl-5,6-dihydro-6H-cyclopenta[b]thiophen-6-one-5-yl)ethylene)-5,5,11,11-tetrakis(4-hexylphenyl)dithieno[2,3-d:2′,3′-d′]-sindaceno[1,2-b:5,6-b′]dithiophene) photoactive layer. Herein, the SFE of TOPD films annealed at different temperatures was quantified jointly from the water contact angle and the diiodomethane contact angle according to the Owens-Wendt (OW) method (Owens and Wendt, 1969). We found that the SFE of TOPD changes as the annealing temperature increases, and the PBDB-T:ITIC-M photoactive layer with the desired vertical component distribution is obtained via fine controlling the SFE of the TOPD layer, leading to a PCE high up to 10.20% for inverted PSCs. The results of time-of-flight secondary-ion mass spectroscopy (TOF-SIMS) visually present the optimized vertical concentration distribution, and the space-charge-limited current (SCLC) method elucidates that the rational vertical component distribution guarantees fully exciton dissociation and facilitates charge transportation.

## Experimental section

### Materials and instrumentations

**Figure 1:**
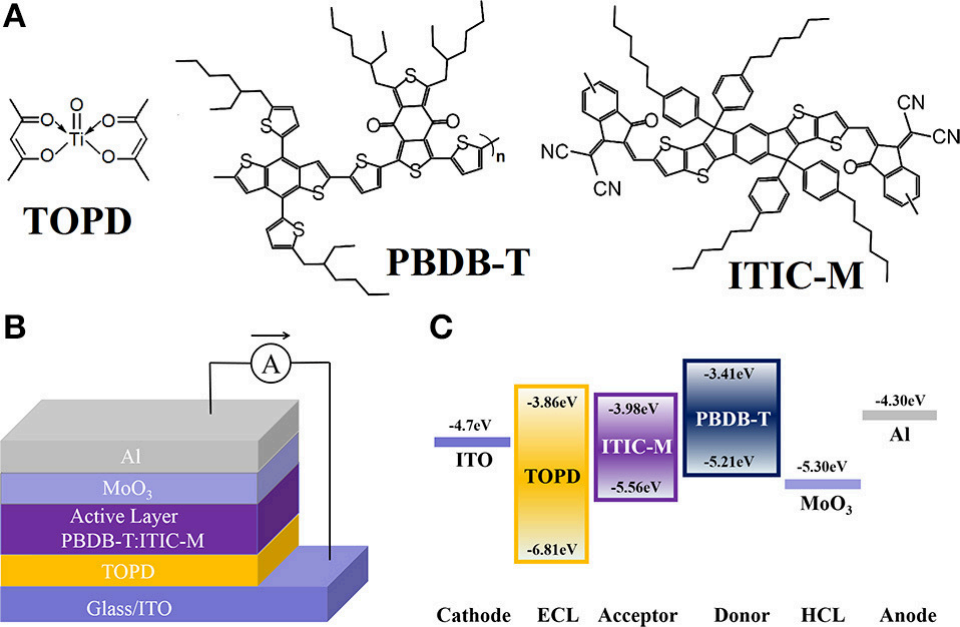
**(A)** Molecular structures of TOPD, PBDB-T, and ITIC-M. **(B)** Architecture of i-PSCs. **(C)** The HOMO and LUMO energy levels of the materials involved in the i-PSCs.

Patterned indium tin oxide (ITO) glass with a sheet resistance of 10 Ω/sq was purchased from CSG HOLDING Co., Ltd. (Shenzhen, China). Both MoO_3_ (purity > 99.0%) and DIO (purity > 98.0%) were purchased from Sigma Aldrich (St. Louis, MO). The TOPD was purchased from Alfa Aesar (Shanghai, China). PBDB-T and ITIC-M were purchased from Solarmer Materials Inc. (Beijing, China), and their molecular structures are displayed in Figure 1A. All these commercially available materials were used as received without further purification.

Ultraviolet–visible (UV-Vis) absorption spectra for TOPD films before and after annealing were measured by a Hitachi U-3010 UV-Vis spectrophotometer. X-ray diffraction (XRD) patterns were recorded on a Siemens D5005 diffractometer using CuKα radiation at 40 kV and 20 mA. The contact angle images were investigated using a profilometer of Dektak XT (Bruker) under ambient conditions. The perpendicular component distribution was analyzed using a TOF-SIMS from ION-TOF GmbH. The Ambios Technology XP-2 surface profilometer was employed to evaluate the film thicknesses involved in the device.

### Device design, fabrication, and characterization

A well-designed device architecture coupled with a desired perpendicular component distribution in photoactive blends is anticipated to prepare efficient PSCs. Figure 1B plots the inverted device structure, where PBDB-T:ITIC-M blend is sandwiched between the TOPD-coated ITO cathode and the high work function (WF) MoO_3_ anode. Figure 1C demonstrates the energy levels of the materials involved in the devices. The PBDB-T:ITIC-M blend absorbs incident photons and produces excitons. The excitons diffuse toward and dissociate at the PBDB-T:ITIC-M interfaces to yield free electrons and holes. The free electrons can breezily transport from the active layer to the cathode through the TOPD layer due to the similar lowest unoccupied molecular orbital (LUMO) of TOPD (−3.86 eV) and ITIC-M (−3.98 eV). Meanwhile, the high up to 1.25 eV hole barrier between the highest occupied molecular orbital (HOMO) level of TOPD (−6.81 eV) and ITIC-M (−5.56 eV) can effectively block the transportation of the hole from PBDB-T to ITO, reducing carrier recombination losses at the interface (Bai et al., 2018). Furthermore, MoO_3_ with WF of 5.30 eV facilitates hole transfer from the active layer to the Al anode (Bai et al., 2017).

All ITO substrates were successively ultrasonically cleaned twice by detergent, deionized water, acetone, and isopropanol. For the regular control device with the architecture of ITO/PEDOT:PSS/PBDB-T:ITIC-M/Ca/Al, the pre-cleaned and dried ITO substrates were treated under UV-ozone (UVO) exposure for 15 min to improve its surface smoothness and WF (Bai et al., 2018). Then a 30 nm PEDOT:PSS layer was deposited by spin-coating its aqueous solution at 2,000 rpm for 45 s, and baked at 150°C for 10 min in air. On the other hand, for the inverted PSCs with the structure of ITO/TOPD/PBDB-T:ITIC-M/Ca/Al, the clean ITO substrates without UVO treatment were transferred into the nitrogen-filled glovebox for the following process. After that, the isopropanol solution with optimized concentrations of TOPD (1,000 rpm/min, 12 mg/mL) were spin-coated on ITO, and finally annealed at different temperatures (80–120°C) for 5 min, and the 14-nm TOPD films with excellent robustness were obtained.

The photosensitive layer was prepared by spin-coating the PBDB-T:ITIC-M chlorobenzene solution (1:1 weight ratio, polymer concentration of 10 mg/mL) with 5% volume ratio of DIO additive on the ITO/PEDOT:PSS and ITO/TOPD substrates at optimized 1,900 rpm for 60 s. Subsequently, the samples were annealed at 100°C for 10 min to obtain the PBDB-T:ITIC-M layer with the thickness of ~100 nm (Bai et al., 2017). Finally, the anode of Ca(10 nm)/Al(100 nm) for the control devices or MoO_3_(24 nm)/Al(100 nm) for the inverted PSCs was thermally deposited on the active layer under a base pressure of 5 × 10^−5^ Pa (Luo et al., 2018).

Device characterization of current density–voltage (*J-V*) performance was conducted in a nitrogen-filled glovebox using a Keithley 2400 Source Measure Unit under simulated AM1.5G solar irradiation with the light intensity of 100 mW/cm^2^ (from SAN-EI LTD, AAA grade). The incident photon to electron conversion efficiency (IPCE) was measured using the QE-R system (Enli Tech., Kaohsiung, Taiwan) in air at room temperature. The intensity of each wavelength both in *J-V* and IPCE was calibrated with the standard single crystalline silicon photovoltaic device purchased from the national renewable energy laboratory. Electron mobility was measured employing the SCLC method for devices with the structure of ITO/Al/TOPD/Al, ITO/Al/PBDB-T:ITIC-M/Al, and ITO/TOPD/PBDB-T:ITIC-M/Al. The results are plotted as ln(*JL*^3^*/V*^2^) vs. (*V/L*)^0.5^. Electron mobility was calculated from the intercept of the corresponding lines on the axis of ln(*JL*^3^*/V*^2^) (Malliaras et al., 1998).

## Results and discussion

### OW method for determination the SFE of solid films

The measurement of the contact angle of sessile drops deposited on different film surfaces is one of the powerful approaches to evaluate the film SFEs and further modulate the vertical concentration distribution of the electron donor and acceptor (Clark et al., 2013). The OW method is the most common approach for polymeric materials so far, in which water and diiodomethane are used. According to OW principal assumptions, the SFE includes dispersion and polar two components. The former represents the dispersion interaction occurring on an interface and the latter is a sum of polar, hydrogen, inductive, and acid–base interactions. The SFE is evaluated with the OW method using the following set of equations (Żenkiewicz, 2007):

(1) $$\begin{array}{l} {\left(\gamma_{Sd}\gamma_{Wd}\right)}^{\frac{1}{2}}+{\left(\gamma_{Sp}\gamma_{Wp}\right)}^{\frac{1}{2}}=0.5\gamma_{W}\left(1+\cos\Theta_{W}\right) \end{array}$$

(2) $$\begin{array}{l} {\left(\gamma_{Sd}\gamma_{Dd}\right)}^{\frac{1}{2}}+{\left(\gamma_{Sp}\gamma_{Dp}\right)}^{\frac{1}{2}}=0.5\gamma_{D}\left(1+\cos\Theta_{D}\right) \end{array}$$

(3) $$\begin{array}{l} \gamma_{S}=\gamma_{Sd}+\gamma_{Sp} \end{array}$$

where W, D, and S represent the polar liquid of water, the dispersion liquid diiodomethane, and the solid film; γ_*s*_, γ_*sd*_, γ_*sp*_ are the SFE, the SFE dispersion component, and the SFE polar component of the solid film; γ_*Wd*_, γ_*Dd*_, γ_*Wp*_, γ_*Dp*_ are the dispersion component and the polar component of water and diiodomethane, and these values are extracted from Owens and Wendt (1969); Θ_*W*_ and Θ_*D*_ are the contact angles of water and diiodomethane, respectively.

**Figure 2:**
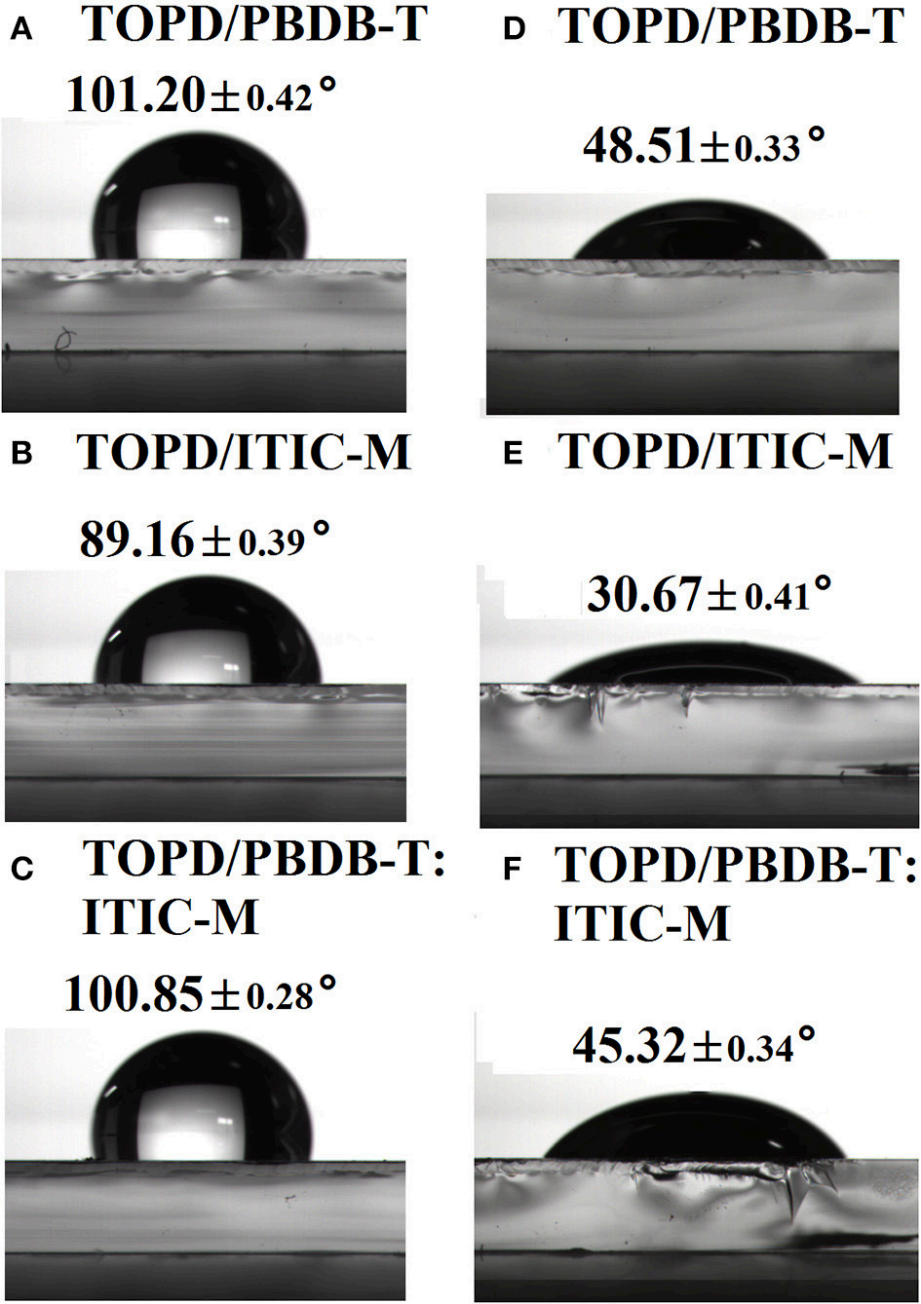
Water contact angle images of **(A)** pure PBDB-T, **(B)** pure ITIC-M, and **(C)** PBDB-T:ITIC-M on the TOPD modified substrate; and diiodomethane contact angle images of **(D)** pure PBDB-T, **(E)** pure ITIC-M, and **(F)** PBDB-T:ITIC-M on the TOPD modified substrate.

Figures 2A–F demonstrate the average water contact angle (WCA) and the diiodomethane contact angle (DCA) of the pure ITIC-M, pure PBDB-T, and PBDB-T:ITIC-M blend films on TOPD-coated ITO. For pure PBDB-T, pure ITIC-M, and PBDB-T:ITIC-M blend film, the WCAs are 101.20° (Figure 2A), 89.16° (Figure 2B), and 100.85° (Figure 2C), respectively; the DCAs are 48.51° (Figure 2D), 30.67° (Figure 2E), and 45.32° (Figure 2F), respectively. Correspondingly, the SFEs for pure PBDB-T and pure ITIC-M are 35.67 and 43.98 mJ/cm^2^ calculated by the OW method, respectively. Apparently, the SFE of PBDB-T is lower than that of ITIC-M, and we can foresee that a much higher proportion of PBDB-T will accumulate at the top surface of PBDB-T:ITIC-M blend film to lower the systematic SFE. As expected, the SFE of the PBDB-T:ITIC-M blend film is 37.56 mJ/m^2^ calculated from the OW method, which visibly indicates that the blend film minimizes its total free energy by increasing the surface concentration of the low-energy component PBDB-T (Jasieniak et al., 2016).

**Figure 3:**
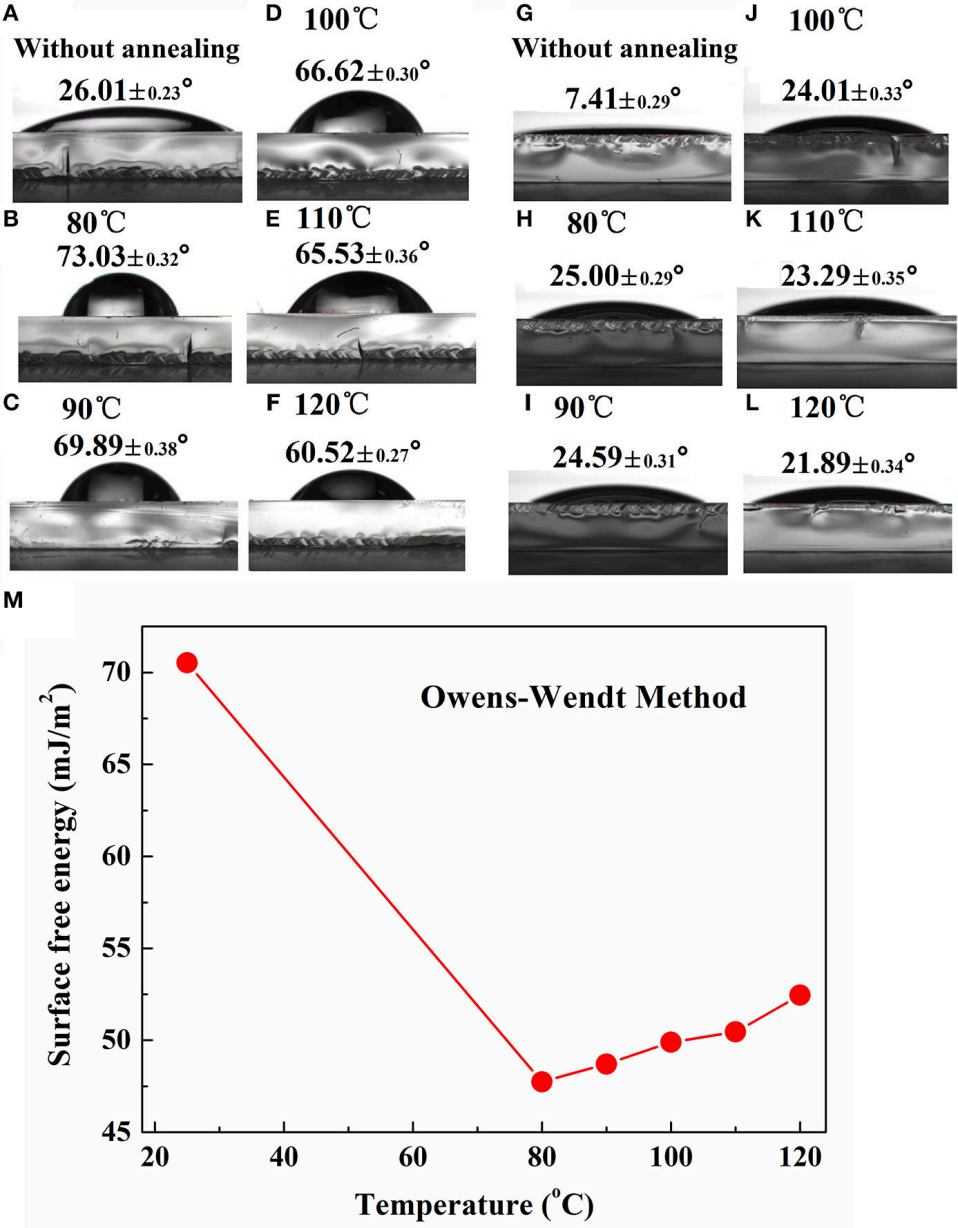
Water contact angle images of TOPD film before **(A)** and after annealing at **(B)** 80°C, **(C)** 90°C, **(D)** 100°C, **(E)** 110°C, and **(F)** 120°C; and diiodomethane contact angle images of TOPD film before **(G)** and after annealing at **(H)** 80°C, **(I)** 90°C, **(J)** 100°C, **(K)** 110°C, and **(L)** 120°C; **(M)** surface free energy of TOPD films before and after annealing calculated from the OW method.

To construct a desired vertical component distribution within PBDB-T:ITIC-M blend film, TOPD was annealed at different temperatures to change its SFE and further modulate the vertical concentration distribution of ITIC-M. Figure 3 presents the WCA and DCA of TOPD films before and after annealing at different temperatures. The WCA and DCA for TOPD film without annealing are only 26.01° (Figure 3A) and 7.41° (Figure 3G), which indicates the high SFE of 70.53 mJ/cm^2^. The WCA for TOPD film annealed at 80, 90 100, 110, and 120 °C are 73.03° (Figure 3B), 69.89° (Figure 3C), 66.62° (Figure 3D), 65.53° (Figure 3E), and 60.52° (Figure 3F); and their corresponding DCA are 25.00° (Figure 3H), 24.59° (Figure 3I), 24.01° (Figure 3J), 23.29° (Figure 3K), and 21.89° (Figure 3L), respectively. The SFEs are 47.74, 48.71, 49.9, 50.46, 52.46 mJ/cm^2^ for TOPD film annealed at 80, 90 100, 110, and 120°C according to the OW method, as shown in Figure 3. Obviously, the SFE increases gradually with the rise in the annealing temperature, and these subtle changes certainly adjust the vertical concentration distribution of ITIC-M.

### Component distribution of PBDB-T:ITIC-M blends at the air surface

**Figure 4:**
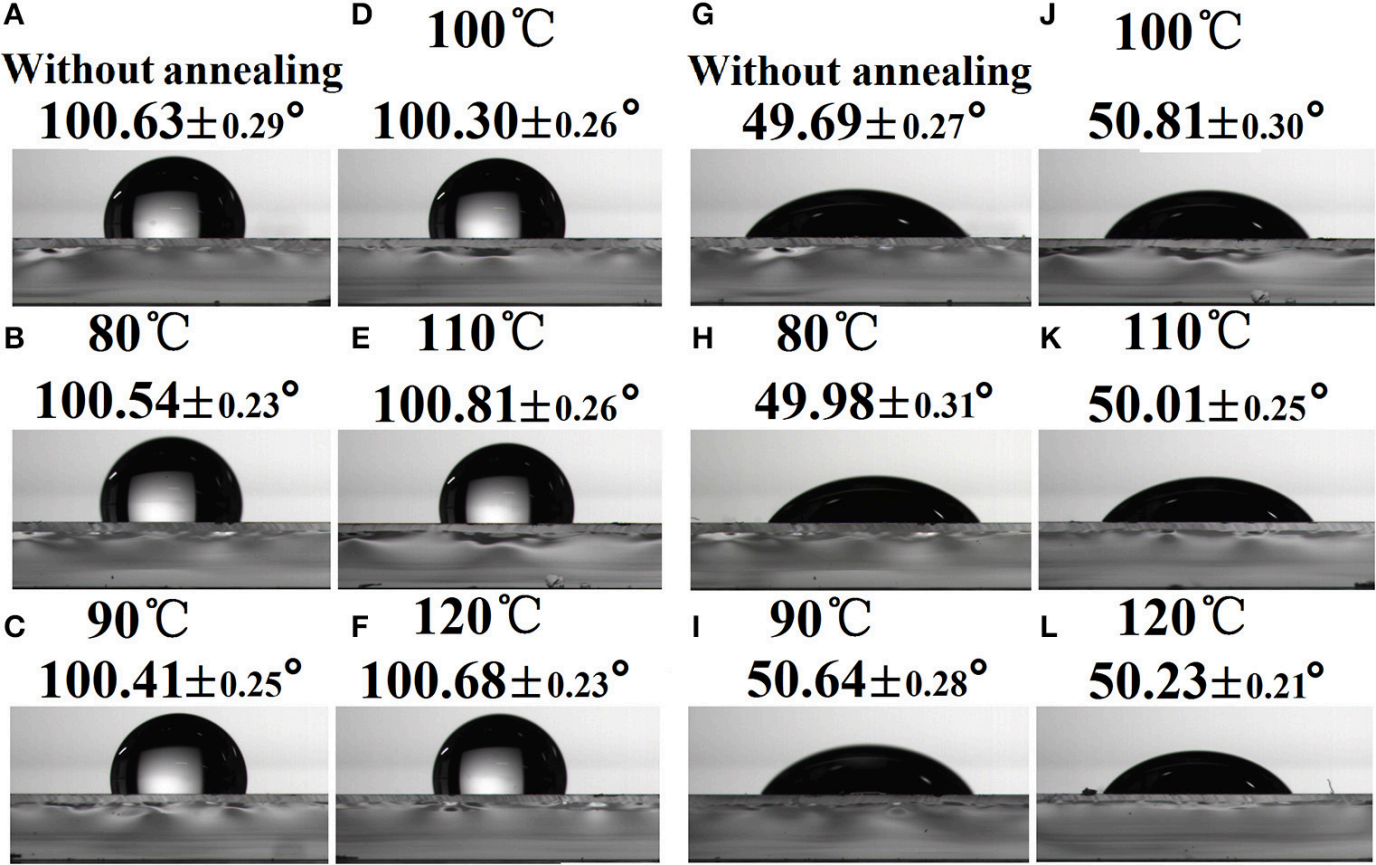
Water contact angle images **(A–F)** and diiodomethane contact angle images **(G–L)** of PBDB-T:ITIC-M on TOPD film without or with annealing.

To clarify the influence of substrate SFEs on the component distribution at the air surface, the WCA and DCA of PBDB-T:ITIC-M on TOPD film without or with annealing are illustrated in Figures 4A-L, respectively. As can be seen, both the WCA and DCA do not strongly depend on substrate SFEs, and their corresponding values remain almost the same whether TOPD is annealed or not. The WCAs change slightly around 100° and the DCAs alter around 50°, which suggests that there is no distinct difference about the component distribution of the electron donor and acceptor at the air surface. Hence, the changes of substrate SFEs hardly affect the component distribution at the top surface region (Björström et al., 2005; Tillack et al., 2011).

### Vertical component distribution within the PBDB-T:ITIC-M photoactive layer

**Figure 5:**
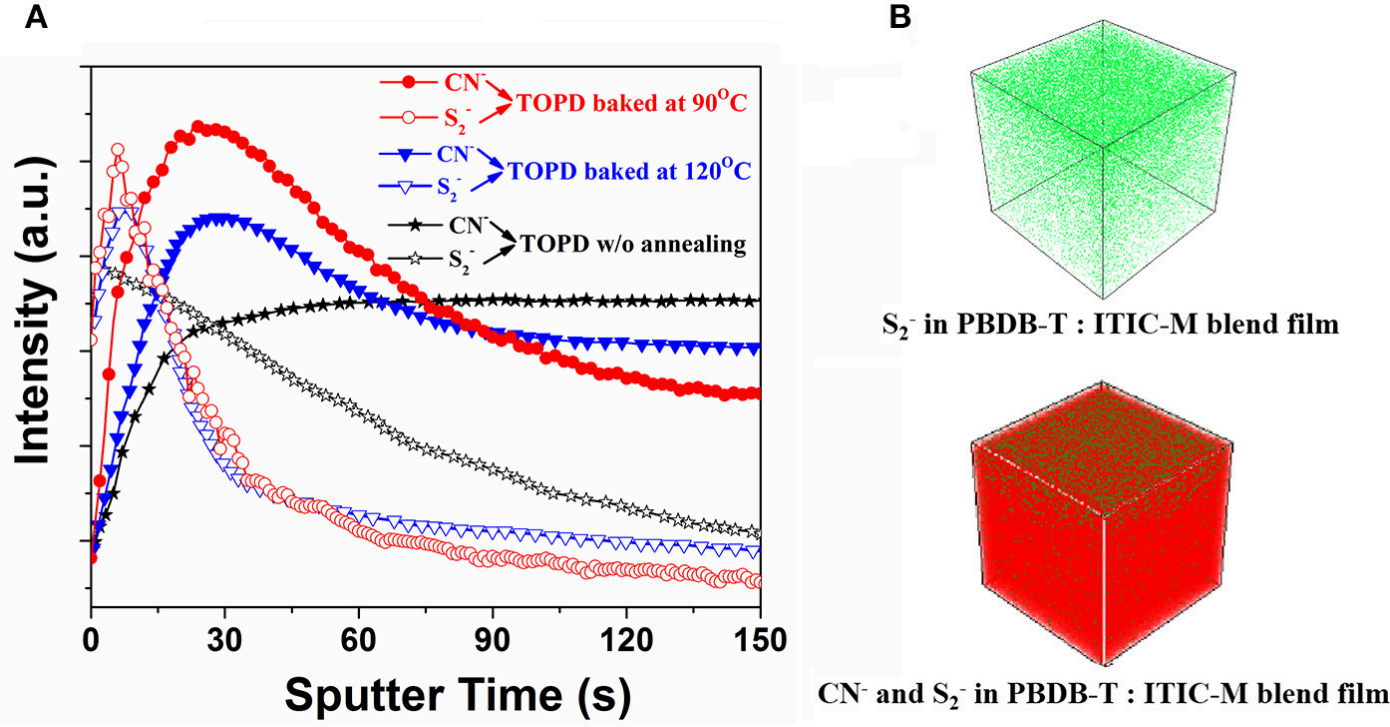
**(A)** ${\text{S}}_{2}^{-}$ and CN^−^ intensity as a function of sputter time. **(B)** Three-dimensional concentration profiles of ${\text{S}}_{2}^{-}$ and CN^−^ in the PBDTBDD:ITIC-M blend films, and the TOPD layer is annealed at 90°C.

The photoactive layers deposited on three typical TOPD cathode buffer layers (before annealing, annealed at 90 and 120°C) were selected to test the TOF-SIMS and further elucidate how SFEs affect the vertical component distribution. Figure 5 plots the intensity change in the signals of ${\text{S}}_{2}^{-}$ and CN^−^ with sputter time since they are characteristic species for PBDB-T and ITIC-M, respectively. As can be seen, the PBDB-T concentration located at the air surface is distinctly higher than that found in the bulk whether TOPD is annealed or not. However, the PBDB-T concentration distribution almost decreases linearly with sputter time for TOPD without annealing; the PBDB-T concentration distribution reaches a maximum value at 6 s for TOPD annealed at 90 and 120°C. When sputter time is beyond 55 s, in increasing order, the PBDB-T concentration distribution value for TOPD annealed at 120°C, next to TOPD baked at 90°C, is lower than that of TOPD before annealing. Obviously, the higher PBDB-T concentration distribution at the substrate interface has a detrimental effect on the collection of carriers for PSCs (Chen et al., 2009). Therefore, it is reasonable to postulate that the PSCs with TOPD baked at 90°C afford high performance.

The distributions of ITIC-M greatly rely on heat treatment. For TOPD film without annealing, the ITIC-M distribution in the blends increases rapidly during the first 30 s and remains constant after 30 s deviating away from the ideal distribution of the electron acceptor. For TOPD film baked at 90 and 120°C, their ITIC-M distributions reach a maximum value at 24 and 30 s, respectively, and the signal intensity of the former is stronger than that of the latter. There is no obvious change for signals of ${\text{S}}_{2}^{-}$ and CN^−^ after the sputter time beyond 150 s. From the results obtained up till now, three aspects have to be addressed. The first is that the air surface is always enriched with a lower-surface-energy polymer component and the influence of substrate SFEs is negligible, which is in accordance with the contact angle measurement results (Tanaka et al., 2010). The second is that the substrate SFEs have a more direct and remarkable effect on the vertical component distribution within the active layer, both for the electron donor and acceptor. Lastly, the TOPD film baked at 90°C with the SFE of 48.71 mJ/cm^2^, which is very close to that of the ITIC film with an SFE of 43.98 mJ/cm^2^, tends to form a desired vertical component distribution facilitating carrier transportaion.

The mechanism of the vertical component distribution is complicated, including thermodynamics, kinetics, surface free energy, and selective dissolubility. In this work, we focus on the SFE of the TOPD layer, which roots from the kinetics of the molecular rearrangement in the blend films (Karagiannidis et al., 2011). As we know, the driving force for the lower SFE constituent accumulation to the high energy surface (air) is the lowering of the overall free energy of the system (Xu et al., 2010), leading to a larger concentration of PBDB-T at the surface. Theoretically, a complete demixing is expected to occur and the formation of a bilayer is thermodynamically possible if the thermal annealing above the polymer's glass transition temperature and the macromolecules obtain the appropriate mobility to rearrange (Klein et al., 2000). However, in the case of the PBDB-T:ITIC-M system, some ITIC-M molecules will probably diffuse into the PBDB-T layer driven by the different surface free energies of TOPD layers to achieve a more thermodynamically favorable component distribution, namely a phase separation will eventually come to a limit (Treat et al., 2011). Hence, the air–film interface is enriched with polymer, while the substrate–film interface is enriched with ITIC-M regardless of the annealing temperature. Films deposited on annealed TOPD show more ITIC-M close to the top surface (higher CN^−^ to ${\text{S}}_{2}^{-}$ signal at around 30 s) induced by the different SFEs of the TOPD layer. Nevertheless, the underlying mechanism of the vertical component distribution is still under way for all researchers, which is also the area warranting our further study.

### Photovoltaic performance and electron mobility

**Figure 6:**
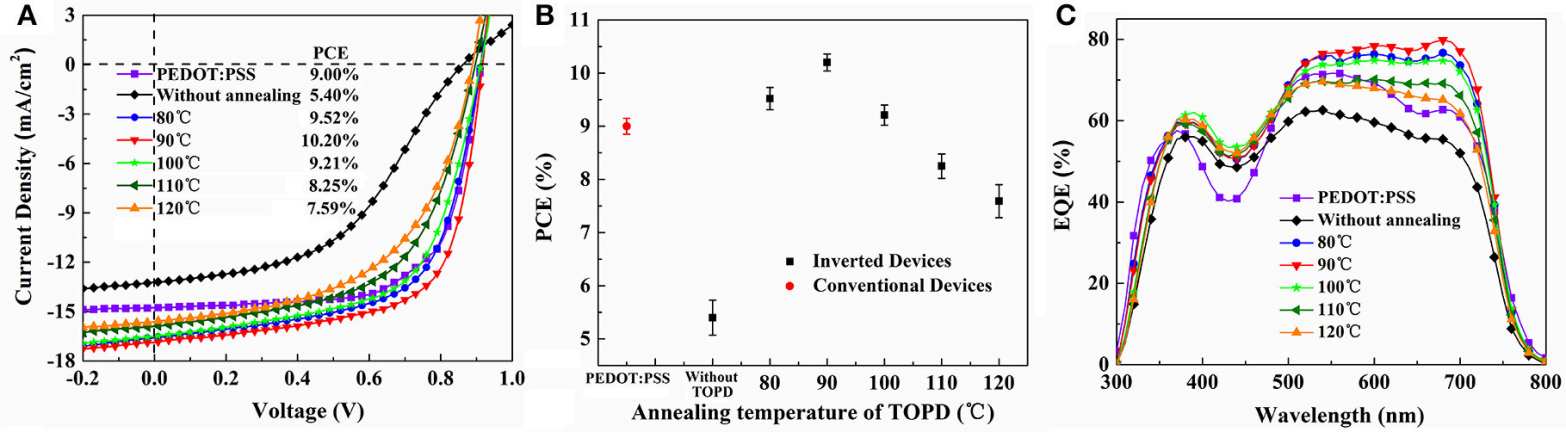
**(A)** *J-V* curves, **(B)** PCE with error bars, and **(C)** IPCE spectra for conventional devices and inverted devices without and with TOPD. The *J-V* curves are measured under the illumination of AM 1.5G at 100 mW/cm^2^.

To further demonstrate the interplay between the vertical component distribution and device performance, *J-V* results, PCE with error bars and IPCE spectra of the control device and i-PSCs with TOPD cathode buffer layers are shown in Figure 6. The key parameters of PCE, short-circuit current density (*J*_*sc*_), open-circuit voltage (*V*_*oc*_), and fill factor (*FF*) under the illumination of AM1.5G, 100 mW/cm^2^ (averaged over 12 individual devices), are compared in Table 1. The control device with PEDOT:PSS shows a PCE of 9.00%, with a *V*_*oc*_ of 0.913 V, a *J*_*sc*_ of 14.76 mA/cm^2^, and an *FF* of 66.78%. The inverted device with an unannealed TOPD layer provides the lowest performance; the PCE, *J*_*sc*_, *V*_*oc*_, and *FF* are 5.40%, 13.21 mA/cm^2^, 0.861 V, and 47.46%, respectivley. After annealing, the four parameters are all enhanced significantly, and the device with TOPD baked at 90°C affords the highest device metrics, with a PCE of 10.20%, a *J*_*sc*_ of 16.88 mA/cm^2^, a *V*_*oc*_ of 0.916 V, and an *FF* of 65.33%. The improvement in *J*_*sc*_ and *FF* benefits from the vertical concentration distribution of PBDB-T and ITIC-M in the active layer, which fortify the charge separation and transportation (Ma et al., 2014). The five annealing temperatures affect PCEs in descending order as 90, 80, 100, 110, 120°C. The results of IPCE in Figure 6B are in agreement with the aforementioned results, which strongly confirm that the vertical concentration distribution of PBDB-T and ITIC-M within the blend film is affected by the substrate SFE greatly.

**Table 1:** Photovoltaic parameters (averaged over 12 individual devices) of the conventional devices and the i-PSCs with and without TOPD under 100 mW/cm^2^.

**Device**	**SFE of TOPD (mJ/cm^2^)**	***V*_oc_ (V)**	***J*_sc_ (mA/cm^2^)**	***FF* (%)**	**PCE (%)**	***R*_s_^a^ (Ω cm^2^)**
Conventional device		0.913	14.76	66.78	9.00 ± 0.15	2.8
Without annealing	70.53	0.861	13.21	47.46	5.40 ± 0.33	6.5
80°C	47.74	0.911	16.57	63.09	9.52 ± 0.21	2.4
90°C	48.71	0.916	16.88	65.33	10.20 ± 0.16	2.1
100°C	49.90	0.912	16.51	61.15	9.21 ± 0.19	3.7
110°C	50.46	0.896	15.89	57.93	8.25 ± 0.23	4.6
120°C	52.46	0.889	15.60	54.67%	7.59 ± 0.31	5.2

a *Series resistance (R_s_) for PSCs in the dark is obtained at 1 V*.

**Figure 7:**
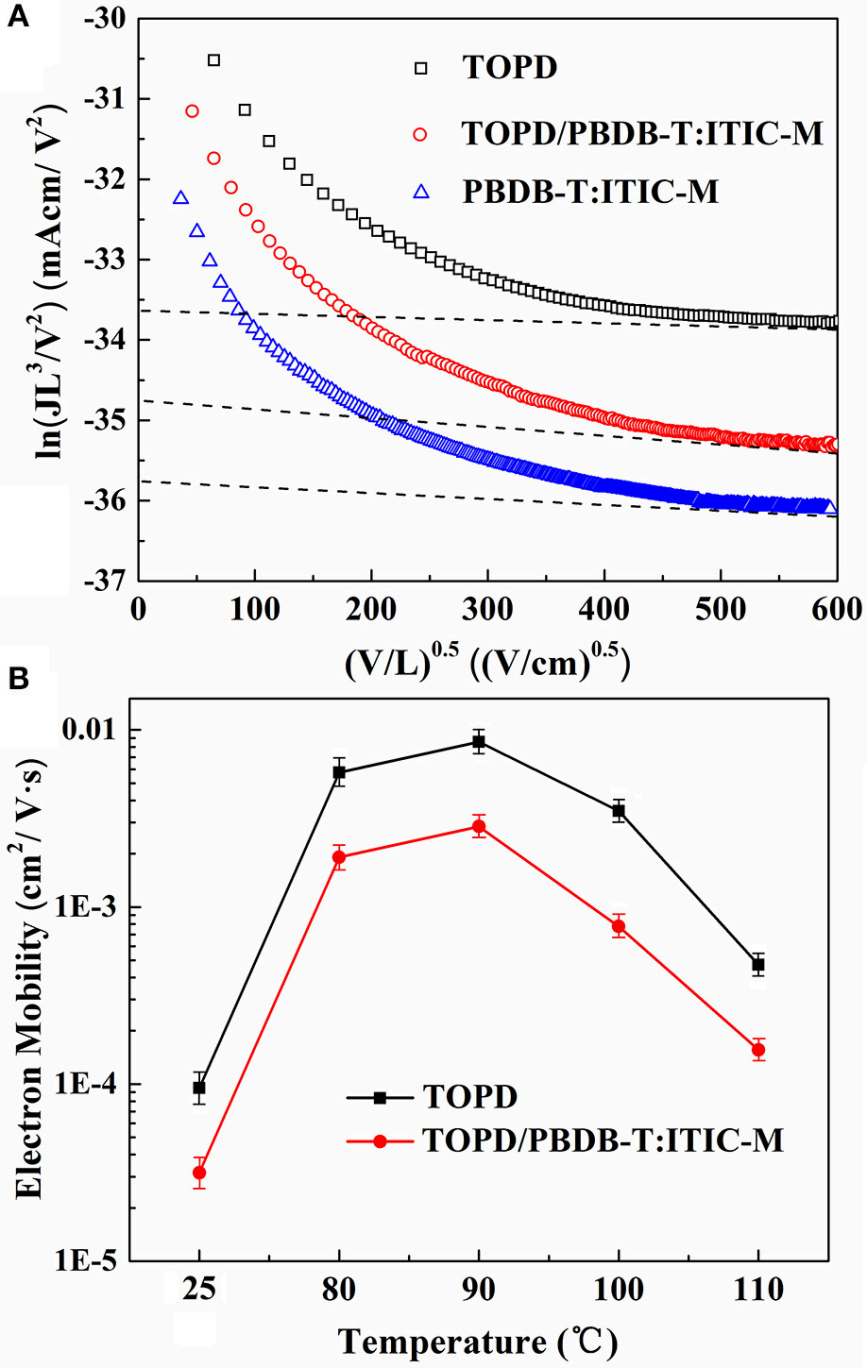
**(A)** *J-V* curves of single-electron devices with the structure of ITO/Al/TOPD/Al, ITO/Al/PBDB-T:ITIC-M/Al, and ITO/TOPD/PBDB-T:ITIC-M/Al; **(B)** the electron mobility for TOPD and TOPD/PBDB-T:ITIC-M as a function of the annealing temperature of TOPD.

To illustrate how charge transportation and collection can be affected in the photoactive blends having different vertical component distributions, the *J-V* curves of single-electron devices (Ahmed and Nakazato, 1996) with the structure of ITO/Al/TOPD/Al, ITO/Al/PBDB-T:ITIC-M/Al, and ITO/TOPD/PBDB-T:ITIC-M/Al are displayed in Figure 7A, in which the TOPD film annealed at 90°C. The electron mobilities of TOPD, PBDB-T:ITIC-M, and TOPD/PBDB-T:ITIC-M are 8.56 × 10^−3^, 1.04 × 10^−3^, and 2.85 × 10^−3^ cm^2^ V^−1^ s^−1^, respectively. The change of the electron mobility as a function of the annealing temperature in Figure 7B shows that TOPD with heat treatment manifests higher charge mobility than that of TOPD film without annealing, and a maximum is passed through. Without annealing, the electron mobilities of TOPD and TOPD/PBDB-T:ITIC-M are 9.51 × 10^−5^ and 3.16 × 10^−5^ cm^2^ V^−1^ s^−1^, respectively. On the other hand, the electron mobilities of TOPD baked at 80, 90, 100, and 110°C are 5.74 × 10^−3^, 8.56 × 10^−3^, 3.48 × 10^−3^, and 4.71 × 10^−4^ cm^2^ V^−1^ s^−1^, and the corresponding electron mobilities of TOPD/PBDB-T:ITIC-M are 1.91 × 10^−3^, 2.85 × 10^−3^, 7.77 × 10^−4^, and 1.56 × 10^−4^ cm^2^ V^−1^ s^−1^, respectively. Therefore, the increased electron mobility for TOPD after heat treatment is in favor of charge transportation and collection. Our results confirm that 90°C is an appropriate annealing temperature for TOPD from the standpoint of charge transportation and collection, which can increase *J*_*sc*_ and *FF* of devices.

### Other structural and optical properties of TOPD films

**Figure 8:**
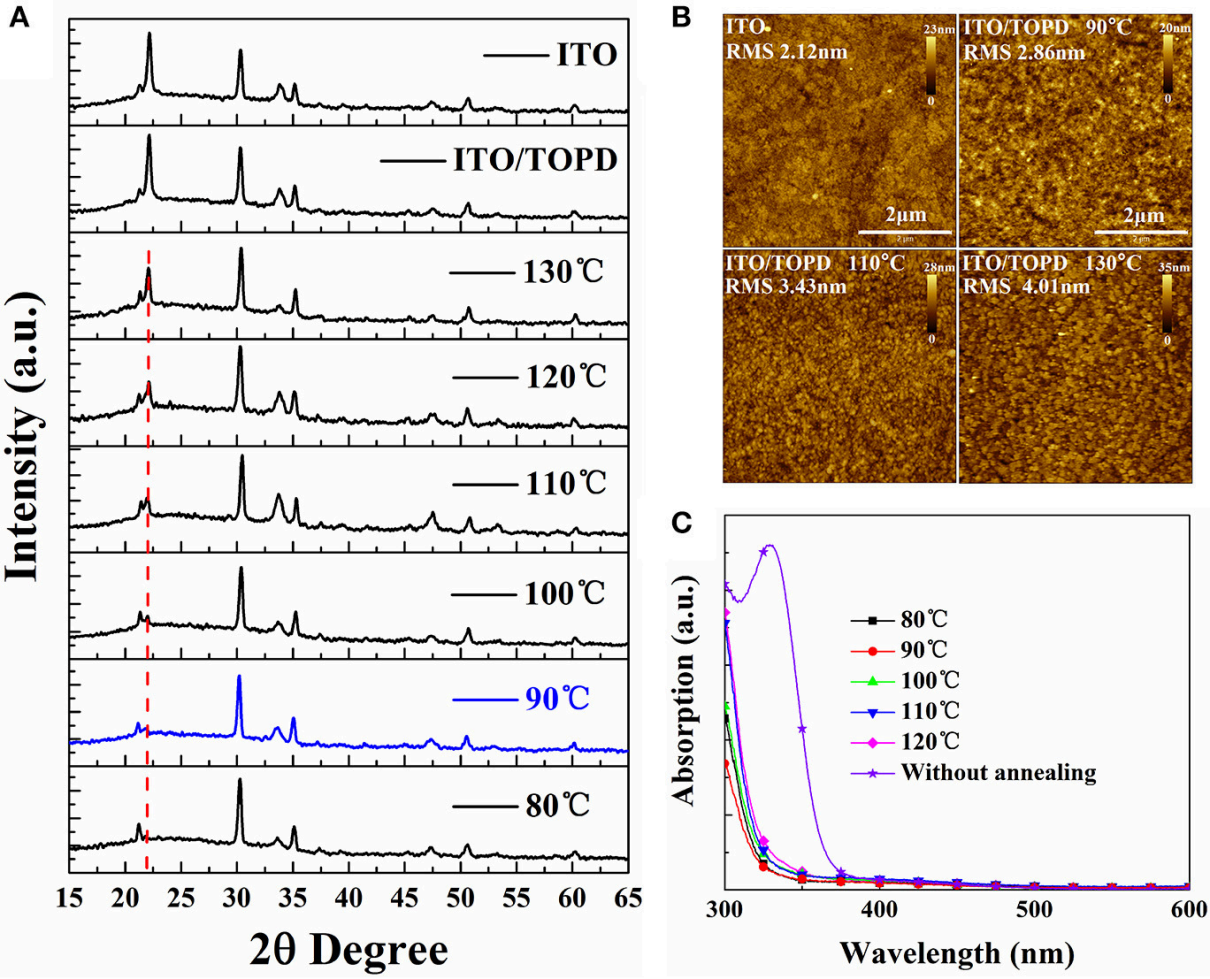
**(A)** XRD patterns, **(B)** AFM images with 5 × 5 μm scan size, and **(C)** absorption spectra of TOPD films on ITO glasses before and after annealing at different temperatures in N_2_ for 5 min.

Other structural and optical information of TOPD film besides the SFE is explored to elaborate the device performance enhancements. Figure 8A shows the XRD patterns for TOPD films before and after annealing. Apparently, there does not appear to be any characteristic peak of TOPD except that of ITO, and all TOPD films are in the amorphous state whether they are annealed or not. But the peak of the glass substrate at 2θ = 22° illustrates obvious changes before and after annealing. Namely, the XRD curve of TOPD/ITO before annealing overlaps well with the bare ITO, and the peak intensity is very strong. On the other hand, there is no obvious peak at 2θ = 22° for TOPD annealed 80 or 90°C, and the peak intensity gradually increases as the annealing temperature increases from 100 to 130°C. This suggests that 80 and 90°C are the suitable temperatures for forming uniform and compact film on the surface of ITO. The different peak intensities for TOPD annealed at higher temperature are caused by different aggregation behaviors of TOPD films. The AFM images of TOPD annealed at different temperatures are shown in Figure 8B. Evidently, the root-mean-square (rms) roughness rises with the increase in the annealing temperature of TOPD, and they are 2.86, 3.43, and 4.01 nm for TOPD annealed at 90, 110, and 130°C, respectively. The suggests that fine and weak aggregation behaviors occur at lower annealing temperature, and the smooth and uniform film formed at lower temperature is favorable for effective charge transport and collection. This is in accordance with the result of the XRD results.

Figure 8C plots the absorption spectra of TOPD films before and after annealing at different temperatures. It is evident that the absorption of TOPD films decreases distinctly after annealing, which is caused by the organic residual solvents because there is no chemical component change before and after thermal annealing (Bai et al., 2018). The TOPD film baked at 90°C demonstrates the weakest absorption, which ensures more light harvesting in the photoactive layer. On the whole, the absorption first decreases slightly as the annealing temperature rises from 80 to 90°C, and then increases as the annealing temperature rises from 90 to 120°C. Therefore, TOPD film baked at 90°C shows superior structural and optical properties in addition to its appropriate SFE.

## Conclusions

Fine-tuning the SFE of the TOPD cathode buffer layer has been explored in this work, with the aim of unraveling the underlying mechanism and rational controlling the vertical distribution of the electron donor and acceptor. Our studies confirm that the SFE of TOPD increases gradually with the rise in the annealing temperature, and these subtle changes certainly cause the profound vertical component distribution within the bulk region of the PBDB-T:ITIC-M. The results of TOF-SIMS visibly demonstrate that TOPD film baked at 90°C with the SFE of 48.71 mJ/cm^2^, which is very close to that of the ITIC film with the SFE of 43.98 mJ/cm^2^, tends to form a desired vertical component distribution facilitating charge transportation. Consequently, compared with conventional BHJ devices without tuning the donor and acceptor concentration, the PCE increases from 9.00 to 10.20% benefiting from the short circuit current density increase from 14.76 to 16.88 mA/cm^2^. The results obtained in this work allow the conclusion that modulation of the SFE of the substrate is a feasible way to control the vertical component distribution of the electron donor and acceptor. This approach holds great potential for practical application of high-efficiency PSCs.

## Author contributions

All authors listed have made a substantial, direct, and intellectual contribution to the work, and approved it for publication.

### Conflict of interest statement

The authors declare that the research was conducted in the absence of any commercial or financial relationships that could be construed as a potential conflict of interest.
